# An outbreak of *Clostridium perfringens* infection on a training ship anchored in Busan, Korea

**DOI:** 10.4178/epih.e2024086

**Published:** 2024-11-07

**Authors:** Seonmi Kim, Hyunjin Son

**Affiliations:** 1 Division of Infectious Disease Control, Yeongdo-gu Public Health Center, Busan, Korea; 2 Department of Preventive Medicine, Dong-A University College of Medicine, Busan, Korea

**Keywords:** *Clostridium perfringens*, Training ship, Food-borne diseases, Outbreak, Korea

## Abstract

**OBJECTIVES:**

In September 2023, an outbreak of food-borne disease occurred among students on a training ship docked in Busan. This was an epidemiological investigation with the aim of improving infection prevention activities and group meal service practices on board ships.

**METHODS:**

In this study, a case was defined as an individual who experienced diarrhea more than twice a day during their training period aboard the training ship. A total of 171 exposed individuals including 6 food handlers was well-defined; therefore, a retrospective cohort study was conducted. We administered a questionnaire and conducted laboratory tests including 38 rectal swab samples. Relative risk (95% confidence interval) for each food item was calculated.

**RESULTS:**

Of the 165 students and school staff members, 41 met the case definition, resulting in an attack rate of 24.8%; all cases were students. The shape of the epidemic curve was unimodal, with the peak from 00:00 to 06:00 on September 7, 2023. *Clostridium perfringens* was detected in 9 cases, and no other pathogens were found. Significant relative risk was shown in 11 different food items.

**CONCLUSIONS:**

*C. perfringens* was the causative pathogen of this outbreak on the training ship. Due to the lack of preserved food samples, the exact source of infection could not be confirmed. Ships are not classified as collective dining facilities, leaving them in a management blind spot. Therefore, specialized guidelines, voluntary inspections by the operating entities, and continuous education for managers and staff are necessary.

## GRAPHICAL ABSTRACT

[Fig f3-epih-46-e2024086]

## Key Message

A food-borne disease outbreak occurred on a training ship in Busan, affecting a significant number of people with *Clostridium perfringens* infection. Among the 165 people, the attack rate was 24.8%, with the epidemic peak occurring between 00:00 and 06:00 on September 7, 2023. This study recommends implementing specialized guidelines, voluntary inspections, and continuous education to improve infection prevention practices on ships, which are not classified as collective dining facilities and thus remain in a management blind spot.

## INTRODUCTION

*Clostridium perfringens* enteritis is an acute gastroenteritis caused by enterotoxins produced by *C. perfringens* proliferation [1]. *C. perfringens* can be present in the intestinal tract of a healthy person or animal, in the natural world (e.g., soil and sewage), and can exist for years as spores in soil [2]. The transmission route is ingestion of undercooked meat (especially poultry), unsterile dairy products, or contaminated water or food. The spores may be present at room temperature, allowing the bacteria to proliferate when stored at room temperature or when improperly heated [3]. If food cooked in large quantities is stored at inappropriate temperatures, an epidemic can occur due to bacterial proliferation. The symptoms of *C. perfringens* food poisoning include severe abdominal cramps and watery diarrhea after a 7-hour to 30-hour incubation period [3,4].

*C. perfringens* is estimated to be the second most common cause of bacterial food-borne illness in the United States, causing one million illnesses each year [1]. *C. perfringens* is also a common cause of food-borne outbreaks. Outbreaks have been reported in a variety of settings across the world and are frequently associated with contaminated cooked meat and poultry dishes, often in mass catering settings [5].

According to the 2023 Korean guideline for control of waterborne and food-borne disease, the number of reported cases of *C. perfringens* infections in 2022 was 1,890, a 40.0% decrease from the previous year (3,151 cases). However, *C. perfringens* infections remain one of the leading causes of food-borne outbreaks, with approximately 10 outbreaks reported annually [6]. In 2022, an epidemiological investigation of a food-borne outbreak reported in a military unit found *C. perfringens* in 8 out of 23 cases. The outbreak was attributed to problems in the preparation and storage process of boxed meals brought in from outside [7].

On September 7, 2023, the supervisor of a training ship docked in Busan reported to the local public health center that several students were complaining of gastrointestinal symptoms (e.g., diarrhea, vomiting). The local public health center, along with the Busan Metropolitan Government and the Busan Center for Infectious Disease Control and Prevention, established an epidemic investigation team and initiated an epidemiological investigation to identify the source of the infection and prevent further spread.

The training ship had been conducting a training program since August 3, 2023, with a total of 171 people on board, including 137 students from A High School, 28 school staff members, and 6 food handlers, and was scheduled to continue until January 31, 2024. The onboard training was conducted according to the Seafarers Act, taking into account the unique nature of maritime labor, and aimed to develop the students’ necessary job performance skills and adaptability by living and working on the ship [8].

## MATERIALS AND METHODS

### Case definition

A case was defined as a student, school staff member, or food handler on the training ship from A High School who experienced diarrhea more than once a day during the training period. In addition, a confirmed case was defined as a case where the causative pathogen, *C. perfringens*, was detected through laboratory tests.

### Study design

The entire group of 171 people on the training ship from A High School was identified as the exposed population, making a retrospective cohort study design appropriate. A questionnaire was administered to all participants to determine the presence of symptoms and the types of food consumed. In addition, detailed questions about external food consumption were asked via telephone to the 41 identified cases. For the six food handlers, an interview was conducted to check for gastrointestinal symptoms, their attire during food preparation, and any injuries on their hands. Since the consumption of an external meal (burger meal) was reported, the food handlers from the burger restaurant were also subjected to the same investigation.

### Laboratory tests

A total of 38 rectal swab samples were collected (30 from cases, 6 from food handlers on the ship, and 2 from food handlers at the burger restaurant). Tests were conducted for 18 types of bacteria and 5 types of viruses that commonly cause gastrointestinal infections. The 18 species of bacteria included cholera, typhoid, paratyphoid, bacterial dysentery, enterohemorrhagic *Escherichia coli*, *Salmonella* species, *Vibrio cholerae*, enterotoxigenic *E. coli*, enteroinvasive *E. coli*, enteropathogenic *E. coli*, *Campylobacter jejuni*, *C. perfringens*, *Yersinia enterocolitica*, *Listeria monocytogenes*, enteroaggregative *E. coli*, and *Vibrio parahaemolyticus*. The 5 types of viruses included group A rotavirus, astrovirus, adenovirus, norovirus, and sapovirus [6].

A total of 17 environmental samples were collected from the galley and cooking utensils of the training ship, drinking water, food ingredients, cooking utensils at the burger restaurant, and a replicated burger meal. These samples were submitted for laboratory testing.

### Statistical analysis

The attack rates for students and staff were calculated separately, with the values representing the number of cases or confirmed cases. The epidemic curve was constructed at 6-hour intervals, considering the incubation period of the causative pathogen, excluding 5 individuals whose exact symptom onset time was unknown. Microsoft Excel 2016 (Microsoft, Redmond, WA, USA) was used to calculate the relative risk (RR) for each food item and drinking water, along with 95% confidence intervals (CIs).

### Ethics statement

This study used data from an epidemiological investigation conducted by the national and local health authorities in accordance with the laws governing situations that require urgent action for public health.

## RESULTS

### Field information

The students on the training ship had been receiving seafarer training since August 3, 2023, either docked along the coast of Busan or moving to offshore locations.

The students lived and ate on the ship, attending regular classes from Monday to Friday. During weekends, when the ship was docked, they were allowed to leave the ship for overnight stays or day trips. Each day, 6–8 school staff and other staff members remained on the ship as duty teachers or workers, while the rest commuted.

The initial symptom onset was on Wednesday, September 6. During the preceding weekend, September 2–3, 112 of the 137 students stayed overnight off the ship. Of the 25 students who remained overnight aboard the ship, some went on day trips one or both days, while some did not go at all. The exact number of students who went on both days was difficult to determine. Students who did not go on trips remained on the ship, attended weekend classes, and consumed meals on board. Except for those who were off the ship due to overnight stays or day trips, all members consumed the same food together on the ship.

**Figure 1:**
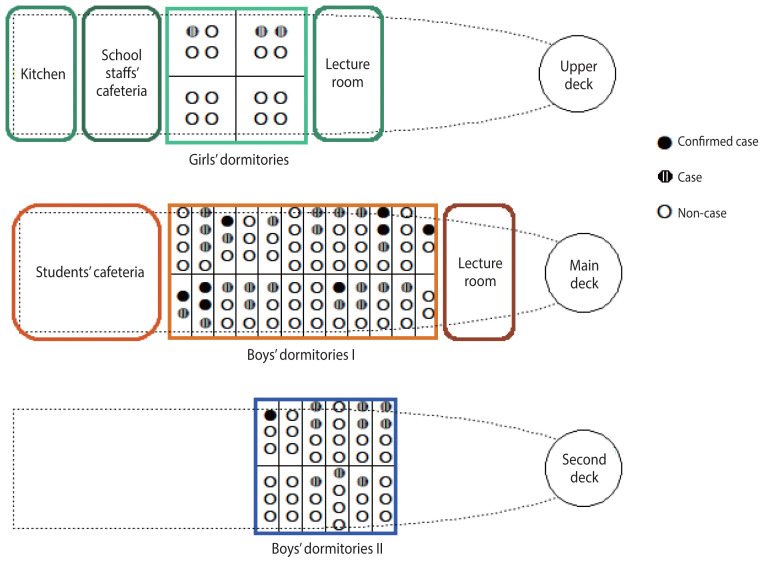
Layout of the main living spaces for students on the training ship.

The students shared 44 dormitory rooms, typically 2 to 4 individuals per room. Each room contained beds, a small wardrobe, and a washbasin (Figure 1). The common areas included classrooms, restrooms, showers, a gym, and a laundry room. The classrooms were also used as dining areas for the students. Classes were usually divided into 2 groups, and the school staff members had their own designated rooms, dispersed in various locations. It was noted that there had been no long-term disembarkations or full disembarkations since the last week of August.

### Environmental investigations

Since the meals were governed by the Seafarers Act, no preserved samples were available. Inspections of the food storage and cooking environments, including the state of food storage, refrigerator and freezer temperatures, and kitchen hygiene, revealed no notable issues. Inspection of the food handlers found no hygiene issues or unusual symptoms, including gastrointestinal symptoms (diarrhea, vomiting) or hand injuries. There were 6 food handlers, with 1 on leave each day. Breakfast was prepared by 2–3 handlers, and 5 handlers prepared both lunch and dinner. Food for both students and staff was prepared in the main kitchen, as shown in Figure 1. All staff members ate lunch in the teachers’ cafeteria, and only 6–8 staff members, including those on duty, ate breakfast and dinner. The students served themselves the prepared meals and ate breakfast, lunch, and dinner in the students’ cafeteria. They were also provided with packaged snacks or cup noodles every other night.

The food ingredients were purchased on August 25 and August 31, prior to the outbreak. Most side dishes were purchased as packaged ready-made products, while other ingredients were cooked and any leftovers were discarded immediately after the meal. During the week leading up to the reported outbreak, the only externally prepared meal that was communally consumed was dinner on Tuesday, September 5. This consisted of a burger meal that included a burger, French fries, and canned cola. According to the meal schedule, breakfast was served from 07:20 to 08:00, lunch from 11:50 to 12:45, dinner from 16:45 to 17:45, and a late-night snack from 21:00 to 22:00.

### Attack rate

The index case that met the case definition first occurred around 19:00 on September 6, 2023. The overall attack rate, excluding food handlers, was 24.8%, with 41 cases out of 165 individuals. Among the students, the attack rate was 29.9%, with 41 cases out of 137 students. All school staff members were asymptomatic, resulting in an attack rate of 0.0%. There were 9 confirmed cases where the causative pathogen was detected, yielding an overall attack rate of 5.5%. The attack rate for confirmed cases among the students was 6.6% (Table 1).

### Epidemic curve

**Figure 2:**
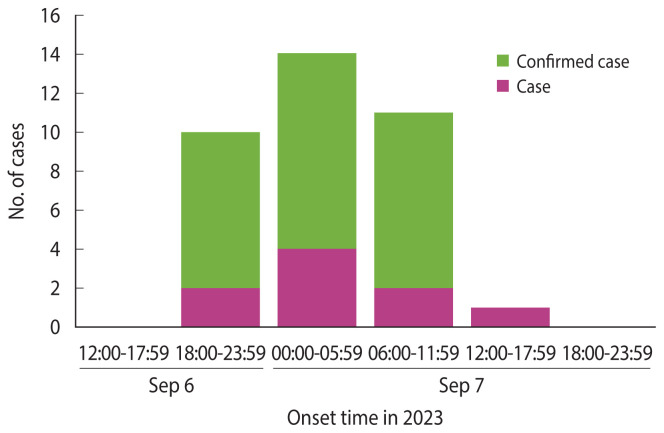
Epidemic curve of 36 cases with known onset time.

Among the 41 individuals that fit the case definition, the index case occurred at 19:00 on September 6 (Wednesday). All cases developed within 24 hours of this initial onset. The shape of the epidemic curve was unimodal, with a peak from 00:00 to 06:00 on September 7. No cases had consumed food other than the communal meals provided. The epidemic curve for the 36 cases with known symptom onset times, excluding the 5 cases with unknown onset times, is shown in Figure 2.

### Frequency of symptoms

All 41 cases experienced diarrhea more than twice a day, which fit the case definition, and 20 individuals (48.8%) also had abdominal pain. Other reported symptoms included fever (n=3, 7.3%), chills (n=7, 17.1%), nausea (n=5, 12.2%), vomiting (n=3, 7.3%), and other symptoms such as heartburn and fatigue (n=2. 4.9%). Among the cases, 8 individuals (19.5%) sought outpatient medical treatment at healthcare facilities or public health centers. There were no hospitalizations or severe cases, and all symptoms improved over time.

### Relative risk of consuming each food item

From September 1 (Friday) to September 6 (Wednesday), the RR and 95% CI were calculated for each food and beverage item commonly consumed by the students, school staff, and food handlers. A food consumption analysis was conducted on 155 subjects, excluding 15 subjects who reported the onset of diarrhea symptoms outside the suspected exposure period, and 1 subject who provided an incomplete response.

A total of 19 meals were provided over the 6 days, including breakfast, lunch, dinner, and snacks, with 117 different food items served. The foods that showed a significant RR are presented in Table 2.

The analysis of commonly consumed foods showed significant RR values for the following items: September 2 (Saturday) breakfast items – fermented fish condiment (RR, 2.29; 95% CI, 1.14 to 4.60) and dried radish (RR, 2.37; 95% CI, 1.10 to 5.10); September 4 (Monday) dinner items – rice (RR, 2.85; 95% CI, 1.20 to 6.80), boneless chicken (RR, 2.76; 95% CI, 1.16 to 6.58), spicy beef soup (*yukgaejang*) (RR, 2.18; 95% CI, 1.04 to 4.56), and tofu (RR, 2.47; 95% CI, 1.22 to 4.97); September 5 (Tuesday) late-night snack – cup noodles (RR, 4.00; 95% CI, 1.89 to 8.46); September 6 (Wednesday) dinner items – rice (RR, 2.68; 95% CI, 1.22 to 5.92), hamburger steak (RR, 3.12; 95% CI, 1.40 to 6.92), frank sausage (RR, 2.91; 95% CI, 1.38 to 6.11), and beef soup (RR, 2.15; 95% CI, 1.07 to 4.28).

### Water investigation results

Investigation of the water revealed that the ship purchased fresh water and stored it in a water tank for use. Drinking water, cooking water, and water for showers were all supplied through lines connected to the tank, with cooking water further filtered through a dedicated purifier. Drinking water was provided using purifiers connected to the ship’s tank (a total of 17 water purifiers), and was consumed using personal water cups and disposable cups. The most recent water purchases were made on August 29 and September 6. The residual chlorine in the drinking water was measured above the standard level. Two drinking water samples were tested and detected no *E. coli*, *Salmonella*, or *Y. enterocolitica* per 250 mL. The residual chlorine concentration results met the drinking water standard of 0.1–4.0 ppm, as measured in 2 sinks and 1 water purifier on the upper deck, 2 sinks and 2 water purifiers on the main deck, and 5 water purifiers throughout the ship. Analysis of the RR for each drinking water source did not yield any significant values.

### Laboratory results

Among the 41 cases, rectal swab specimens were collected from 30 individuals, and *C. perfringens* was detected in 9 of them. No other pathogens were found. Of the 6 food handlers required to provide specimens, *C. perfringens* was detected in 2 asymptomatic individuals. No pathogens were detected in the 2 food handlers from the burger restaurant, which provided the external meal.

A total of 17 environmental samples were collected, including 7 samples of remaining food ingredients, 3 samples of a replicated burger meal from the external food service, 5 samples from cooking utensils, and 2 samples from the drinking water of the primary purifiers. No viruses or bacteria were detected in these samples.

### Control measures

Personal hygiene education was conducted for the students and school staff members. It was found that partially cooked frozen foods were stored at room temperature for more than 3–4 hours after being thawed and cooked in large quantities before serving. Food hygiene education was provided to the food handlers to address this issue. Food handlers were also instructed to be more thorough in hygiene practices during food preparation and handling. Until the outbreak was resolved, environmental disinfection was carried out at least twice a day, and any additional cases were to be reported immediately. and only fully cooked meals were served.

## DISCUSSION

Since *C. perfringens* was detected in 9 cases, it can be inferred that *C. perfringens* was the causative pathogen of the gastrointestinal infection outbreak on the training ship. Diarrhea and abdominal pain were the main symptoms and generally resolved within 1 day. The clustering of cases within 1 day on the epidemic curve aligns with the short incubation period characteristic of *C. perfringens*. Furthermore, the unimodal epidemic curve indicated that the outbreak was due to a single common exposure. Considering the incubation period of *C. perfringens*, the food served during lunch or dinner on September 6 was suspected as the infection source. However, due to the nature of the ship, there was no obligation to preserve food samples, making it impossible to conduct an investigation and laboratory testing on the food. Analysis of the RR for each food item showed high RRs for the foods served at dinner on September 6, suggesting that these foods were the likely sources of infection.

The investigation into the layout of educational and training spaces, as well as the dormitory rooms, did not show any spatial clustering of cases. Considering that *C. perfringens* is rarely transmitted person-to-person and primarily causes infection through contaminated food, it cannot be ruled out that the gastrointestinal infection outbreak resulted from undercooking or improper storage temperature during mass food preparation.

During the investigation, there were limitations in collecting meaningful human specimens as some staff members were reluctant to provide samples or report symptoms. Initial confusion in the investigation arose due to students visiting clinics for non-symptomatic reasons in order to leave the ship. However, after this initial confusion, the students and staff actively cooperated with the epidemiological investigation, significantly aiding this study.

Due to the periodic departure and docking of the training ship, it was difficult to implement control measures. However, thanks to the active cooperation of the training ship personnel, no additional cases occurred.

The presence of large groups of people on ships with limited sanitation, as well as poor food supplies, leads to an increased risk of infectious diseases [9]. Moreover, the working spaces on ships are narrow, making it difficult to maintain distance, densities are high during meals, and ventilation is poor [10]. These environments promote the spread of infectious diseases, potentially infecting vulnerable groups. Hygienic environments on ships are important to ensure the health and well-being of the people on board [11]. Therefore, it is important to educate everyone on food hygiene and verify their understanding of food safety [12].

In this investigation, specimen testing results from the cases suggested *C. perfringens* as the causative pathogen. However, due to the lack of preserved food samples, the exact source of infection could not be confirmed. The ship was not obligated to preserve food samples because it was not classified as a group meal facility. In addition, most of the ingredients from previous meals had been consumed, so the food that the cases had ingested could not be sampled. During this investigation, only the remaining ingredients available on the day of the investigation were collected and tested.

Ships are not classified as group meal facilities and operate under their own regulations, placing them in a management blind spot [13]. Therefore, specialized guidelines, voluntary inspections by the operating entities, and continuous education for managers and staff are necessary. In particular, preparing large quantities of food for large groups and exposing it to room temperature for extended periods increases the risk of food-borne disease outbreaks [14]. Furthermore, even when food is reheated, a significant number of *C. perfringens* bacteria may survive despite exposure to time and temperature [15].

In this investigation, partially cooked frozen foods were stored at room temperature for more than 3–4 hours after thawing and cooked in large quantities before serving. This highlights a need for standardized guidelines and measures to actively educate food handlers on the correct methods for storage of ingredients before and after cooking, proper cooking methods, and adherence to personal hygiene rules [16,17]. In addition, it is essential to educate the ship’s crew members about food hygiene and safety [18].

This investigation highlighted the unique situation of training ships as temporary group meal facilities. To eliminate the blind spot in infectious disease management on training ships, health authorities and relevant stakeholders should recognize training ships as an extension of schools and seek appropriate methods to manage the food services provided for trainees at the same level as school students.

## Figures and Tables

**Figure f3-epih-46-e2024086:**
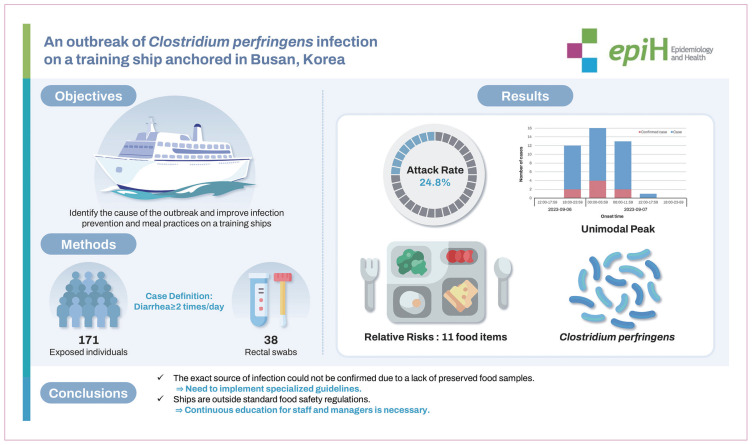


**Table 1:** Attack rates (ARs) of *Clostridium perfingens* among students and school staff

Category	Exposed individuals (n)	Case (AR)	Confirmed case (AR)
Total	165	41 (24.8)	9 (5.5)
Students	137	41 (29.9)	9 (6.6)
School staff	28	0 (0.0)	0 (0.0)

Values are presented as number (%).

**Table 2:** The menu items showing significant RRs for infection in the meals served aboard a training ship

Date	Food item	Attack rate (%)		RR (95% CI)
		Ingestion group^1^	Non-ingestion group^2^	
Sep 2 (Sat) Breakfast (07:20–08:00)	Salted fish	57.1 (4/7)	25.0 (37/148)	2.29 (1.14, 4.60)
	Dried radish	60.0 (3/5)	25.3 (38/150)	2.37 (1.10, 5.10)
Sep 4 (Mon) Dinner (16:45–17:45)	Rice	32.4 (36/111)	11.4 (5/44)	2.85 (1.20, 6.80)
	Boneless chicken	32.1 (36/112)	11.6 (5/43)	2.76 (1.16, 6.58)
	Spicy beef soup	31.8 (34/107)	14.6 (7/48)	2.18 (1.04, 4.56)
	Tofu	34.0 (33/97)	13.8 (8/58)	2.47 (1.22, 4.97)
Sep 5 (Tue) Late-night snack (21:00–22:00)	Cup noodles	40.0 (34/85)	10.0 (7/70)	4.00 (1.89, 8.46)
Sep 6 (Wed) Dinner (16:45–17:45)	Rice	34.3 (37/108)	12.8 (6/47)	2.68 (1.22, 5.92)
	Hamburger steak	35.3 (36/102)	11.3 (6/53)	3.12 (1.40, 6.92)
	Frank sausage	35.7 (35/98)	12.3 (7/57)	2.91 (1.38, 6.11)
	Beef soup	33.7 (35/104)	15.7 (8/51)	2.15 (1.07, 4.28)

RR, relative risk; CI, confidence interval.

1 Number of cases/number of people who ingested the food item.

2 Number of cases/number of people who did not ingest the food item.
